# The genotypes and virulence attributes of *C. albicans* isolates from oral leukoplakia

**DOI:** 10.4317/medoral.24748

**Published:** 2021-05-23

**Authors:** Manjula Manoji Weerasekera, Gayan Kanchana Wijesinghe, Asanga Sampath, Ayomi Dilhari, Thilina Madhumal, Rasika Dilrukshi, Rajitha Willaddara, Sunil Karunathilaka, Chinthika Gunasekara, Neluka Fernando, Lakshman Samaranayake

**Affiliations:** 1 Department of Microbiology, Faculty of Medical Sciences, University of Sri Jayewardenepura, Gangodawila, Nugegoda, Sri Lanka; 2 Department of Basic Sciences, Faculty of Allied Health Sciences, University of Sri Jayewardenepura, Gangodawila, Nugegoda, Sri Lanka; 3 Department of Chemistry, Faculty of Science, University of Colombo, Kumaratunga Munidasa Mawatha, Colombo 07, Sri Lanka; 4 Oral and Maxillofacial Unit, Colombo South Teaching Hospital, Kalubowila, Dehiwala, Sri Lanka; 5 College of Dental Medicine, University of Sharjah, UAE; 6 Faculty of Dentistry, University of Hong Kong, Hong Kong

## Abstract

**Background:**

There is a debate as to whether some types of oral leucoplakias (OL) are caused by *Candida* species, and whether they contribute to the malignant transformation, associated with a minority of such lesions. As no detailed population analysis of yeast isolates from OL is available, we evaluated the virulence attributes, and genotypes of 35 *C. albicans* from OL, and compared their genotypes with 18 oral isolates from healthy individuals.

**Material and Methods:**

The virulence traits evaluated were esterase, phospholipase, proteinase, haemolysin and coagulase production, and phenotypic switching activity, and yeast adherence and biofilm formation. DNA from OL and control yeasts were evaluated for A, B or C genotype status.

**Results:**

Phospholipase, proteinase, and coagulase activity and biofilm formation was observed in 80%, 66%, 97 % and 77 % of the isolates, respectively. Phenotypic switching was detected in 8.6%, while heamolytic, and esterase activity and adherence were noted in all isolates.

**Conclusions:**

The genotype A was predominant amongst both the OL and control groups. Due to the small sample size of our study a larger investigation to define the role of candidal virulent attributes in the pathogenicity of OL is warranted, and the current data should serve as a basis until then.

** Key words:**C. albicans, oral cavity, leukoplakia, virulence factors, genotyping.

## Introduction

Candida species are recognized as important opportunistic pathogens of humans (1). Although non-albicans *Candida* species are increasingly identified both in health and disease, *Candida albicans*, with a battery of key virulence attributes, reigns supreme as the predominant human yeast pathogen. The attributes of *Candida* species that assist their survival within the adverse human oral ecosystem include their ability to adhere to epithelia and develop biofilms, the ability to secrete asparty (l) proteinases, phospholipases, haemolysin, esterases, and coagulases (2-5). There is also a profound diversity in expression of these virulence attributes amongst pathogenic *C. albicans* strains which is thought to affect the outcome of infection (6).

C. albicans isolates can be can be classified in several ways, and one such distinctive method is the genotyping of the yeasts based on their 25S rDNA. Accordingly, three major genotypes, A, B. and C (7), and two minor, rare variants D and E (8) have been identified. Several studies report a predominance of Genotype A in oral disease (9) while others have noted significant positive associations between *C. albicans* genotypes, and different oral pathologies (10).

Candida-associated oral leukoplakia (OL) (literally, a white patch), also known as chronic hyperplastic candidiasis, is a relatively common ailment amongst older populations (11-12). *Candida*-associated oral leukoplakia has been associated with increased malignant potential compared to non-Candida leukoplakia, and up to 10 to 15 per cent of the lesions progress into malignancy, if untreated. It is thought that the chronic inflammatory response of the epithelium evoked by various noxious enzymes of the yeast play a key role in the malignant transformation process (13). In addition, Abdulrahim *et al* [2013] have reported a significant association of Genotype C with *Candida* leukoplakia (1). However, the specific role, if any, of the different *C. albicans* genotypes in the pathogenesis of OL is yet to be determined.

Hence the aim of this study was to investigate a battery of eight key virulence determinants of the *C. albicans*, from OL patients, and to correlate them with the demographic variables of the group. We also took the opportunity to genotype yeasts form OL patients and a healthy control group without leukoplakia.

## Material and Methods

- Study design and study setting

A case control study was carried out at the Oral and Maxillofacial clinic of the Colombo South Teaching Hospital, and at the Department of Microbiology, University of Sri Jayewardenepura, Sri Lanka. The study protocol was approved by the Ethics Review Committee of University of Sri Jayewardenepura, Sri Lanka (Ref: MLS 2014/06) and Colombo South Teaching Hospital, Sri Lanka (Ref: 344). Further, the patient approval was also sought and obtained prior to the sample, and data collection.

- *Candida* isolation and speciation

A total of 80 oral swab specimens were collected from the patients with clinically diagnosed oral leukoplakia, and processed as previously described (12). Out of total of 80 specimens, 36 *Candida* isolates were presumptively identified as *C. albicans*, based on the germ tube test, corn meal agar test, and growth at 42 0C on Sabouraud Dextrose Agar (SDA). Of these 35/36 were confirmed as *C. albicans* using duplex PCR – RFLP and were used in the present study.

Similarly, 18 isolates which were isolated from oral rinse specimens of healthy volunteers, as described by Samaranayake *et al* [1986] and were confirmed as *C. albicans* using duplex PCR – RFLP were used as controls for the genotyping study (14).

The *Candida* strains including *C. albicans* (ATCC 10231), *Candida* tropicalis (ATCC 13803), **Candida* parapsilosis* (ATCC 22019), *Candida* glabarata (ATCC 90030) and *Candida* dubliniensis (ATCC MYA 580) were used as the internal standard/reference strains for comparison of the virulence attributes.

- DNA extraction

The genomic DNA was extracted from the *Candida* isolates using the previously published conventional bead beater–phenol chloroform extraction method (15-16). In brief, a loopful of isolated *C. albicans* colonies was suspended in 100 µl STES buffer (200 mM Tris-HCl (pH 7.6), 100 mM EDTA (Ethylenediaminetetraacetic acid), 0.1% SDS (Sodium Dodecyl Sulfate)) and 40 µl of TE (Tris-EDTA) buffer (10 mM Tris-HCl (pH 8), 1 mM EDTA), 120 µl Phenol: Chloroform mixture (1:1 V/V) and 0.3 g sterile zirconium beads (0.1 mm diameter; Bio Spec-Products) were added. The suspension was homogenized using a mini bead beater (model 3110BX; Bio Spec Products) at 480 rpm (26 x g) for 5 min. Hundred microliter from the upper aqueous phase was transferred to a sterile micro centrifuge tube, and DNA was allowed to be precipitated in the presence of 220 µl cold absolute ethanol and 10 µl of 3 M sodium acetate at -20 ºC for 18 h. The solution was centrifuged at 13000 rpm (18928 x g) for 12 min. DNA pellet was air dried and dissolved in 30 µl TE buffer. Extracted DNA samples were quantified using NANO drop 2000/200C spectrophotometer (Thermo Fisher Scientific, USA) and stored at -20 ºC until used.

- Duplex PCR

Duplex PCR amplification was carried out in order to identify and exclude any of other *Candida* species present among presumptively identified 36 of *C. albicans* isolates. PCR amplification was carried out with a final volume of 25μl: each reaction with 2μl of template DNA, 1x PCR buffer (Sigma), 3.0mM of MgCl2 (Promega), 0.2mM of each deoxynucleotide triphosphates (dNTPs) (Promega), 0.5µl of 1μg/μl Bovine Serum Albumin (BSA) (Promega), 1.25U of Taq Polymerase (Sigma-Aldrich, St. Louis, MO, USA) and 0.2µM of each 4 primers, which were previously published universal fungal primers, including ITS1 (5’-TCC GTA GGT GAA CCT GCG G-3’) and ITS4 (5’-TCC TCC GCT TAT TGA TAT GC-3’) which targeted the ITS region of *Candida* Species and previously published INT1 (5’-AAGTATTTGGGAGAAGGGAAAGGG-3’) and INT2 (5’-AAAATGGGCATTAAGGAAAAGAGC-3’) primers which amplify a part of CaYST1 gene intron of *C. albicans*.

PCR amplification was done using GeneAmp PCR systems 9700 (Applied Bio Systems, USA). An initial denaturation step at 94°C was followed by 30 cycles of denaturation at 94°C for 30s, annealing at 54°C for 30s and extension at 72°C for 40s, and the final extension step was 72°C for 10 min. Resulted PCR product were separated and visualized by 1.5% (w/v) agarose gel, stained with ethidium bromide (0.5µg/ml) (Sigma) at 80V for 40 minutes, in TBE (90mM Tris, 90mM Boric acid, 2mM EDTA) (Promega) buffer and visualized using a UV trans-illuminator (Vilber Lourmat, QUANTUM ST4).

- Genotyping

PCR amplification was carried out in a final volume of 25μl of each reaction with 0.2µM of each primers CA-INT-L (5’-ATA AGG GAA GTC GGC AAA ATA GAT CCG TAA-3’) and CA-INT-R (5’-CCT TGG CTG TGG TTT CGC TAG ATA GTAGAT-3’) which targeted the transposable intron in the 25S rDNA. PCR was done with the initial denaturation at 94°C followed by 35 cycles of denaturation at 94°C for 45s, annealing at 57°C for 30s and extension at 72°C for 1min, and the final extension at 72°C for 10min. PCR products were separated using a 1.5% (w/v) agarose gel, as described above and visualized by a UV trans-illuminator.

- Determination of virulence factors

Phospholipase activity: In vitro phospholipase activity of *C. albicans* was detected by using the egg yolk agar plate assay. Size of precipitation zone was measured after growth at 37 oC for up to 4 days as described by Samaranayake *et al* in 1984 (17).

Proteinase activity: In vitro proteinase activity of *C. albicans* was analyzed based on bovine serum albumin (BSA) degradation. The incubation period was up to 5 days at 37oC. Inoculated bovine serum albumin agar plates were stained with Amido black after incubation and unstained area around colonies denotes protein degradation.

Haemolysin activity: Haemolysin activity of *C. albicans* was detected by using Sabouraud’s dextrose agar supplemented with 3% glucose and human blood (pH=5.6) method.

Seven milliliters of fresh human blood was added to 100ml SDA supplemented with 3% glucose. Inoculated blood agar plates were incubated at 37oC for 48 hours in 5% CO2. Addition of human blood instead of sheep blood was the modification.

Esterase activity: Esterase activity of each *C. albicans* isolate was performed by using Tween 80 opacity test medium as described by Aktas *et al* (2).

Coagulase activity: Coagulase activity of each *C. albicans* isolate was determined by using EDTA rabbit plasma method described by Yigit *et al* 2008 (3).

All of the assays described above were carried out in triplicate in three different occasions for all isolates, and results were expressed as the mean of the triplicate.

Biofilm formation assay: The ability of biofilm formation of *C. albicans* was detected by the tube method. A loopful of fresh culture isolates were inoculated into sterile pre labeled screw capped conical polystyrene tubes containing 10ml of Sabouraud dextrose broth supplemented with glucose at a final concentration of 8%. The tubes were loosely capped and incubated at 37oC for 48h. After 48 hours of incubation, the broths in tubes were aspirated out gently using a pasteur pipette. The tubes were washed with distilled water for two times and stained with 1% safranin for 10 minutes. Each isolate was tested in triplicate. The results were interpreted by two observers independently.

Adherence to human buccal epithelial cells: Human buccal epithelial cells (BEC) were collected from five healthy human volunteers and fresh BECs were used for the assay as described by Tsang *et al*. in 1999. BEC suspension was made in PBS and adjusted to a concentration of 1×105 cells/ml using a haemocytometer. *Candida* cells were suspended in PBS and adjusted to a concentration of 1×107 cells/ml using a haemocytometer (18).

Phenotypic switching: *Candida* cell suspensions containing a total of 0.5×103 - 1×103 cells/ml were prepared in sterile normal saline, using standard dilutions to get about 50-100 Colony Forming Unit (CFU)/plate. By using a sterile glass spreader 0.1ml of above prepared cell suspension was spread on YPD (Yeast Extract Peptone Dextrose) agar containing 5mg/L Phloxin B and incubated for 5 days at 25 oC. The colonies with phonotypic switching were counted.

- Statistical analysis

Statistical analysis was carried out using 17.0 version of SPSS software. The chi-square test (X2 test) and Fisher’s exact test were carried out to assess possible association. All these tests were two-sided. The level of significance was specified as *p* < 0.05.

## Results

- Demographic data

Of the total 35 oral leukoplakia patients 31 were males and 4 were females. Mean age of the study group was 60 years (range 42 and 86 years). A total of 18 controls were included (10 males and 8 females) with a mean age of 58 years (range 41 to 71 years). As for the demographics of the OL patients, 27/35 (77.1%) were smokers, while 30/35 (85.7%) were betel nut chewers, and 23/35 (65.7 %) were habitual alcohol consumers.

- Genotypes

**Figure 1:**
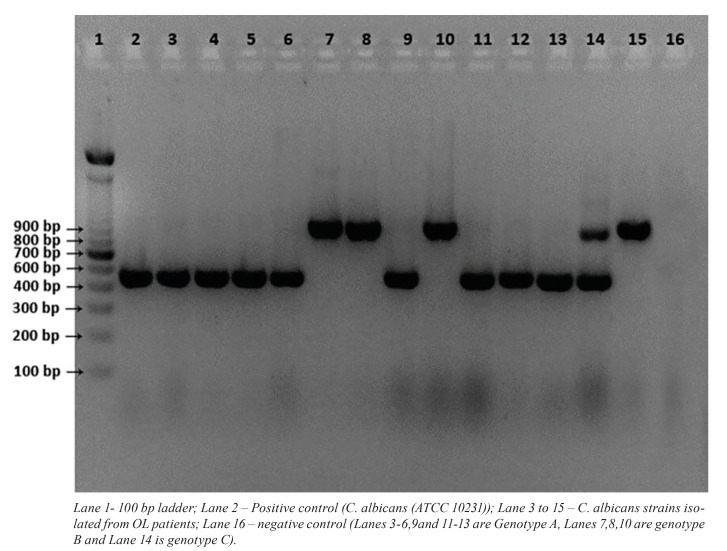
A representative gel electrophoresis image showing the ABC genotypes of 13 of 35 <italic>C. albicans</italic> isolates from oral leukoplakia patients.

Amplifying a target region of the transposable intron in the 25S rDNA by CA-INT-L and CA-INT-R differentiated *C. albicans* into three genotypes: A, B and C. This method yielded a single band each for genotype A (450 bp) and B (840 bp), and two bands for genotype C (450 and 840 bp) (Fig. 1). Of the 35 *C. albicans* isolates from OL patients 21 and 13 were found to be genotypes A and B, respectively; only a single isolate belonged to genotype C. Amongst isolates form healthy individuals, 14 were genotype A, and two isolates each were genotype B and C (Table 1).

When the association of *C. albicans* ABC genotypes of OL isolates were correlated with respective to their gender and smoking habits, alcohol consumption and betel-quid chewing, no significant association was noted between the above variables and the genotypes (*p*> 0.05; Table 2).

- Virulence attributes

The selected eight virulence attributes of *C. albicans* from OL patients were cross tabulated against their genotype, gender and habits including smoking, alcohol consumption, and betel-quid chewing (Table 3). In general, there was no significant association between all eight virulence attributes of the yeasts evaluated, and the foregoing demographic variables (*p*>0.05; Table 3).

Phospholipase activity: Phospholipase activity was detected in 80% (28/35) of *C. albicans* isolates from OL patients. They were predominantly high phospholipase producers with an activity score of 4+ (Table 4) with a mean phospholipase activity of 0.429 ± 0.034 (range 0.36 - 0.48). The mean phospholipase activity for the control strain of *C. albicans* ATCC 10231, was 0.39.

Of the phospholipase positive *C. albicans* isolates, 71.42% (20/28) were from smokers, 64.28% (18/28) were from alcohol abusers, and 78.57% (22/28) were from betel chewers. Even though results indicated that the mean phospholipase activity was higher among smokers, alcohol users and betel chewers, than the counterparts who abstained from these habits (controls) there was no significant association between the phospholipase activity and the habits, nor the gender of the cohort nor the genotype of the *C. albicans* isolates (*p* > 0.05).

**Table 1:**
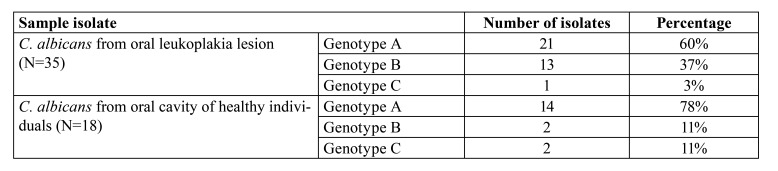
Genotype variation among <italic>C. albicans</italic> isolated from oral leukoplakia patients and healthy individuals.

**Table 2:**
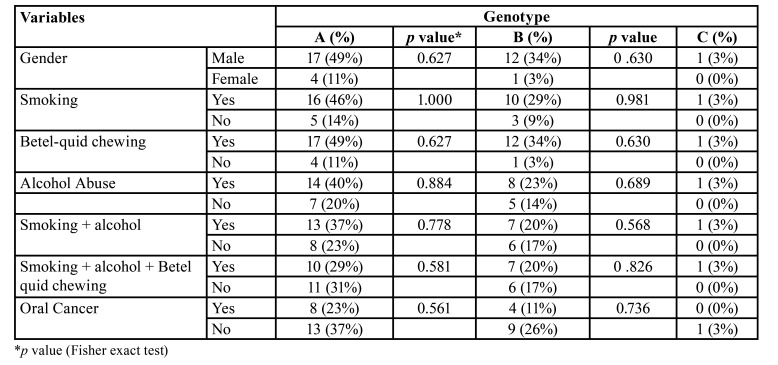
The relationship of <italic>C. albicans</italic> genotypes from oral leukoplakia patients and their demographic characteristics.

**Table 3:**
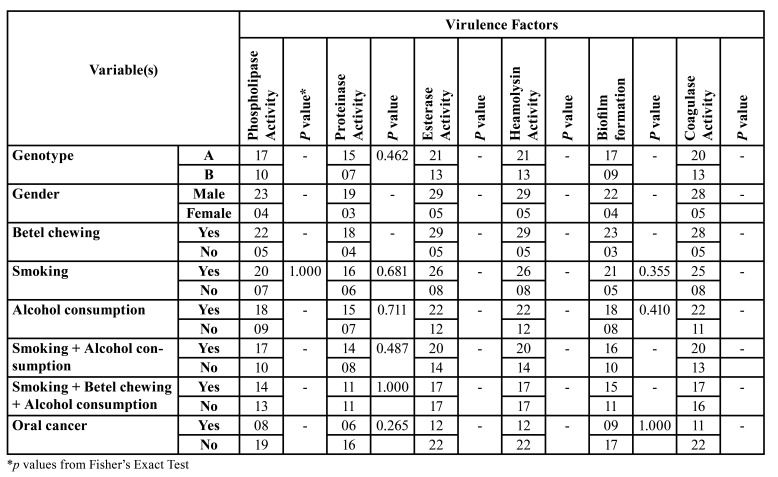
The relationship between the virulence factors of <italic>C. albicans</italic> genotypes and the patient demographics.

**Table 4:**
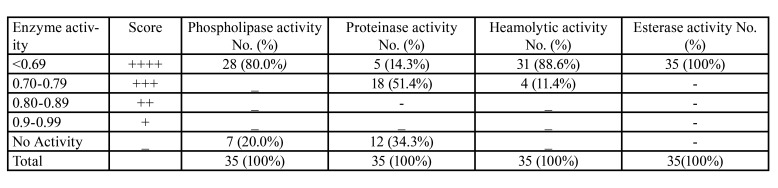
Phospholipase, proteinase, heamolytic and esterase activity of 35 oral <italic>C. albicans</italic> isolates form oral leukoplakia patients.

Proteinase activity: Of the 35 *C. albicans* isolates from OL patients, 23 (65.71%) showed proteinase activity, out of which 21.74% (5/23) demonstrated strong proteinase activity with a 4+ score. A moderate proteinase activity, with a 3+ score was observed in 78.26% (18/23) isolates, while 52.17% (12/23) had no discernible activity. The mean proteinase activity of 35 isolates was 0.721 ± 0.076 (range 0.51 - 0.78.) while this Figure was 0.62 for the control strain *C. albicans* ATCC 10231.

Of the proteinase positive *C. albicans* isolates, 69.56% (16/23) were from smokers, 65.22% (15/23) from alcohol users, and 78.26% (18/23) from betel chewers. As in the case of phospholipase activity no significant association was seen between proteinase activity, and the genotype of *C. albicans* isolates, nor the gender, nor the betel chewing habit of the cohort (*p* > 0.05) (Table 4).

Haemolysin activity: All tested isolates from OL patients were found to be haemolysin producers, with 88.6% (31/35) having strong activity (4+ score) and 11.4% (4/35) with a moderate activity (3+ score). Heamolytic activity ranged between 0.52 - 0.73 with a mean of 0.623 ±0.053 (range 0.52 - 0.73). The mean haemolysin activity of the control *C. albicans* ATCC 90028 was 0.66 (Table 4).

Of the haemolysin positive isolates 77.14% (27/35) were from smokers, 65.71% (23/35) were from alcohol abusers, and 85.71% (30/35) were from betel chewers. Heamolytic activity was higher among smokers, alcohol users and betel chewers than non-users, although no significant association was noted between the latter habits and the yeast heamolytic activity (*p*>0.05; Table 4).

Esterase activity: All of the tested *C. albicans* isolates from OL patients had the ability to produce opaque halos around their colonies on Tween 80 opacity test medium within 3 days of incubation at 370C. All isolates were strong esterase producers having a 4+ score, with a mean score of 0.347 ± 0.023 (range 0.29 - 0.39). The mean esterase production score for the control *C. albicans* ATCC 10231 was 0.34 (Table 4). Among esterase positive *C. albicans* isolates, 77.14% (27/35) were from OL patients who were smokers, 65.71% (23/35) were from alcohol abusers, and 85.71% (30/35) from betel chewers. However, there was no significant association between the esterase activity of the isolates and the latter habits (*p* > 0.05).

Coagulase activity: Out of 35 isolates, 34 (97.14%) *C. albicans* isolates from OL patients were positive for coagulase activity in EDTA rabbit plasma after 24h incubation at 370C. Of the coagulase positive isolates 76.47% (26/34) were isolated from OL patients who were smokers, and 67.65% (23/34) were from alcohol users, and 85.29% (29/34) were from betel chewers. However, there was no significant association between the esterase activity and the latter habits (*p* > 0.05). As in the case of esterase activity there was no significant association between the coagulase activity of the isolates, and the latter habits (*p* > 0.05). The summary of phospholipase, proteinase, heamolysin, esterase and coagulase activity of 35 *C. albicans* isolates are provided in Table 4.

Biofilm formation: Biofilm formation using the tube method was detected in a majority 77.14% (27/35) of *C. albicans* isolates from OL patients. Among biofilm producers, 14.8% (4/27) isolates demonstrated strong biofilm growth with a score of 3+ while 18.5% (5/27) had moderate activity (score 2+), and two thirds (18/27; 66.7%) expressed weak activity (score 1+). Of the biofilm forming *C. albicans* isolates, 81.48% (22/27) were isolated from OL patients who had a smoking habit, and 70.37% (19/27) were alcohol abusers and 88.89% (24/27) were betel chewers. Although not statistically significant biofilm formation was higher among patients who had the habits of smoking, alcohol consumption and betel chewing (*p*>0.05).

Adherence assay: All the tested *C. albicans* isolates from OL patients had the ability to adhere to human buccal epithelial cells *in vitro*. The mean number of adherent yeast cells per 100 buccal epithelial cells was 689.06 ± 245.05 and ranged between 140-980. Of the isolates which had the ability to adhere to host buccal epithelial cells, 77.14% (27/35) were from patients who were smokers, 65.71% (23/35) were from alcohol abusers and 85.71% (30/35) were from betel chewers. The adhesion of *C. albicans* to human BEC appeared to be higher in isolates form patients who were smokers, alcohol abusers, and betel chewers, although not to a significant extent (*p*>0.05).

Phenotypic switching: Cellular morphology of switched white/opaque yeast cells was confirmed using light microscopy, after staining with Lacto phenol cotton blue. White cells appeared as spherical and bright, whereas opaque cells appeared as oval and dark.

White to opaque phenotypic switching was detected only in a small minority of 8.6% (3/35) *C. albicans* isolates form OL patients. All of the phenotypically switched *C. albicans* isolates were from smokers, alcohol users and betel chewers. However, there was no significant association between the latter habits and switching of the isolates (*p*>0.05).

## Discussion

Candida - associated oral leukoplakia is a relatively common ailment with significant potential for malignant transformation, with upto 15% of chronic lesions becoming dysplastic if not managed properly (11-12). *C. albicans* shows an array of virulence factors including the ability to adhere to, and form biofilms on human epithelia, and to produce a wide range of proteolytic enzymes (2-5). These attributes are thought to play a key role in the pathogenesis of OL although very few, if any, have studied them in a collection of *C. albicans* isolates from these lesions. Hence, we comprehensibly evaluated the expression of eight important virulence attributes of *Candida* including extracellular hydrolytic enzyme production, biofilm formation, adherence and phenotypic switching of yeast isolates derived from OL lesions.

In general, all *C. albicans* isolates exhibited virulence attributes to varying degrees, whilst some strains exhibited all the evaluated attributes, the others did so relatively inconsistently. It is well known that the expression of *C. albicans* virulence vary depending on the disease conditions and the related confounding factors (19-20).

An essential prerequisite for successful colonization and establishment of candidal infection within the oral cavity, is the ability of the yeasts to adhere to the host epithelium (4). Adherence to mucosae impedes the dislodgement of yeasts due to the physical flushing action of saliva, and thereby help the pathogen persistence. Therefore, it is not surprising that all yeast isolates exhibited this attribute to varying degrees, a trait that would be crucial for survival on the leukoplakic epithelium. Surface adhesion of yeasts *in vivo* is immediately followed by biofilm formation and in the case of candidal leukoplakia, epithelial penetration, to gain a firm foothold for survival within the hostile oral ecosystem. An over whelming, 77 per cent of our isolates from OL lesions produced biofilms, a feature that has been noted by others who recorded the biofilm forming potency of *C. albicans*, ranging between 50-100% (21-22).

Once the yeasts colonize a mucosal surface they produce extracellular enzymes that inevitably generate an inflammatory host response and epithelial proliferation, resulting in the leukoplakia (13). The extracellular hydrolytic enzymes such as proteinases, phospholipases, esterase, haemolysin and coagulase may also promote foacl breaches of the host epithelial barrier, and facilitate tissue invasion (4,23). Once again all our isolates produced all six of the studied extracellular enzymes to varying degrees, although no single isolate could be discerned as more virulent, in terms of enzyme production, than its counterparts. This observation is not surprising as yeasts need a raft of such attributes to survive on the host epithelium and cause tissue damage.

Heamolytic activity, for instance, was detected in all oral *C. albicans* isolates. Haemolysin contributes to the pathogenicity of *C. albicans* (23-24) as it helps yeast survival within the host through acquisition of iron from host erythrocytes (24). In addition, esterase activity was also detected in all oral *C. albicans* isolates studied. Esterases hydrolyze lipid compounds of host cells membranes, thereby causing cell dysfunction and disruption, and it is likely they facilitate *Candida* adherence and tissue penetration. Finally, we noted coagulase activity in all except a single *C. albicans* isolate. The latter, a little studied hydrolytic enzyme of *Candida*, is also known to damage host cells in-vivo (3,25).

The white/opaque switching mechanism of *Candida*, which entails changing the antigenic composition of its cell walls, is known to help the yeast to evade host defenses (26). This phenomenon was rarely expressed in our isolates, as only 3/36 (8.6%) were such `switchers`. As the reported rates of phenotypic switching varies depending on the site and type of fungal infection (27), it is tempting to speculate that switching attributes of the yeasts may not be critical for epithelial colonization, in OL.

We also correlated the virulence attributes and the demographic factors of the host and noted that the putative virulence attributes of *C. albicans* were expressed to a higher degree in isolates from smokers/betel chewers, than those who had no such habits, although not to a significant extent, possibly due to the small sample size. In general, smokers present with a more acidic saliva with a reduced buffering capacity, and under these conditions, yeast proteinases and phospholipases tend to be more active (17,28-29). Furthermore, smoking favor *Candida* infections by increasing oxidative stress and release of pro-inflammatory factors, and suppressing immune mechanism of the oral cavity. This may possibly explain higher yeast prevalence amongst smokers. Indeed, it is well established that smokers, in general, harbor more oral yeasts than non-smokers.

In terms of the genotypes of the studied isolates, a majority, 60% of our isolates belonged to the genotype A. Others have also confirmed the predominance of the latter genotype, in oral candidal infections (10,25,29). On the contrary, Abdulrahim *et al* (2013) have recently reported a significant association of Genotype C with *Candida* leukoplakia (10). Nevertheless, in other systemic candidal infections Genotype A was the most invasive sub-type, akin to blood stream isolates (30). We were, however, unable to elicit an association between the genotype and the examined virulence attributes of the strains, and this could be due to the small sample size. Further work with a larger number of isolates from OL patients and healthy controls are required to confirm or refute our findings.

## Conclusions

C. albicans isolates form OL patients exhibited a range of virulence attributes such as adhesion to epithelial cells, biofilm formation, and the expression of hydrolytic enzymes (phospholipase, proteinase, haemolysin, esterase, and coagulase) suggesting that they play a crucial role in the pathogenesis of OL. *C. albicans* genotype A was the commonest genotype. Hence a large scale study to understand the precise role, if any, of the candidal virulent attributes in the pathogenicity of OL, and their association with the host demographics is warranted. The current information, in the absence of other similar data, should serve as a basis for future studies.
